# Characterization of Single-Spheroid Oxygen Consumption Using a Microfluidic Platform and Fluorescence Lifetime Imaging Microscopy

**DOI:** 10.3390/bios14020096

**Published:** 2024-02-11

**Authors:** Santhosh Kannan, Chien-Chung Peng, Hsiao-Mei Wu, Yi-Chung Tung

**Affiliations:** 1 Research Center for Applied Sciences, Academia Sinica, Taipei 115201, Taiwan; santhoshk@gate.sinica.edu.tw (S.K.); vp@gate.sinica.edu.tw (C.-C.P.); 2 Department of Engineering and System Science, National Tsing Hua University, Hsinchu 300044, Taiwan; 3 Nano Science and Technology Program, Taiwan International Graduate Program (TIGP), Academia Sinica, Taipei 115201, Taiwan; 4 Department of Biomechatronics Engineering, National Taiwan University, Taipei 106319, Taiwan; hsmwu@ntu.edu.tw; 5 College of Engineering, Chang Gung University, Taoyuan 33302, Taiwan

**Keywords:** spheroid, oxygen consumption, microfluidics, frequency domain fluorescence lifetime imaging microscopy (FD-FLIM)

## Abstract

Oxygen consumption has been used to evaluate various cellular activities. In addition, three-dimensional (3D) spheroids have been broadly exploited as advanced in vitro cell models for various biomedical studies due to their capability of mimicking 3D in vivo microenvironments and cell arrangements. However, monitoring the oxygen consumption of live 3D spheroids poses challenges because existing invasive methods cause structural and cell damage. In contrast, optical methods using fluorescence labeling and microscopy are non-invasive, but they suffer from technical limitations like high cost, tedious procedures, and poor signal-to-noise ratios. To address these challenges, we developed a microfluidic platform for uniform-sized spheroid formation, handling, and culture. The platform is further integrated with widefield frequency domain fluorescence lifetime imaging microscopy (FD-FLIM) to efficiently characterize the lifetime of an oxygen-sensitive dye filling the platform for oxygen consumption characterization. In the experiments, osteosarcoma (MG-63) cells are exploited as the spheroid model and for the oxygen consumption analysis. The results demonstrate the functionality of the developed approach and show the accurate characterization of the oxygen consumption of the spheroids in response to drug treatments. The developed approach possesses great potential to advance spheroid metabolism studies with single-spheroid resolution and high sensitivity.

## 1. Introduction

Oxygen consumption and metabolic profiling are fundamental aspects of cellular behavior, physiology, and the manifestation of various diseases, including neurodegenerative disorders and cancers [1]. Cells rely on a continuous supply of oxygen to meet their energy demands and sustain essential cellular functions. Metabolic alterations, encompassing changes in glycolysis, oxidative phosphorylation, and nutrient utilization, are intricately linked to the availability of oxygen in the cellular microenvironment. The dysregulation of oxygen tension and metabolic pathways can have profound impacts on cell behavior, proliferation, differentiation, and responses to external stimuli, including therapeutic interventions [2,3].

Conventional monolayer cell culture systems have historically served as the foundation for investigating cellular physiology and responses to external stimuli. However, these two-dimensional (2D) models often fall short of reproducing the complexity and heterogeneity of the in vivo cellular microenvironment. In recent years, there has been a growing recognition of the significance of three-dimensional (3D) cell cultures, such as spheroids or organoids, as a more physiologically relevant platform for investigating cellular behavior and responses [4]. In 3D cultures, cells self-assemble into multicellular aggregates, forming spatially organized structures that closely mimic the architecture and physiology of tissues and tumors in vivo.

The metabolic profiling of cells within 3D cultures has been the focus of increasing attention due to its potential to yield insights into the dynamic metabolic processes occurring within complex cellular systems. The ability to characterize the metabolic profiles of 3D cultures provides valuable information regarding nutrient availability, oxygen consumption, and the accumulation of waste metabolites within the microenvironment. Such profiling enables a deeper understanding of cellular adaptation, metabolic plasticity, and their response to therapeutic interventions, all of which are essential for the development of effective strategies for drug treatment [4,5].

In 3D cell culture, studying single spheroids presents a unique opportunity to investigate the influence of spheroid size and heterogeneity on cellular behavior and metabolic responses [6]. However, a significant challenge has been the absence of suitable techniques and devices that enable the cultivation and non-invasive monitoring of individual spheroids in a controlled manner. Existing methods often involve complex procedures or fail to provide real-time insights into the dynamics of oxygen tension within spheroids.

The monitoring of oxygen tension in live 3D spheroids is especially challenging due to their sensitivity to physical damage and susceptibility to a loss of structural integrity during measurements and exposure to ambient oxygen. While there have been recent advancements in techniques like microcomputed tomography [7], electron parametric resonance oximetry [8], electrochemical measurement-based immunohistochemical labeling [9], microelectrode-based probes [10,11], and other clinical approaches [12], these methods are invasive and likely to introduce undesired cytotoxicity and cellular damage throughout the observation. Recently, non-invasive optical approaches utilizing fluorescent labeling and microscopy techniques for oxygen sensing have shown their promise in analyzing 3D spheroid samples [13,14,15]. However, optical sensors often rely on color changes or fluorescence intensity measurements, making them sensitive to ambient light and susceptible to variations due to sample concentrations and photon scattering artifacts, which can result in inconsistent measurements [16].

In response to these challenges, we developed a microfluidic platform specifically designed to form, handle, and culture uniform-sized spheroids. The platform is integrated with widefield frequency domain fluorescence lifetime imaging microscopy (FD-FLIM) for consistent oxygen sensing in this study [16,17]. In our approach, the microfluidic device facilitates the formation and culture of spheroids within microwells using straightforward instrumentation and operations. The cell suspension solution is then replaced with one containing an oxygen-sensitive fluorescence dye, and the spheroids are isolated by covering the microwells with oil. Oxygen tension variations surrounding individual spheroids within the microwells can then be characterized in a time-lapsed manner.

The widefield FD-FLIM system not only provides high-throughput capabilities within a conventional fluorescence microscope setup but also ensures the accuracy of measurements. Furthermore, the reduction in light exposure on the cells and the sensing dyes help mitigate photocytotoxicity and photobleaching, respectively, making time-lapsed measurements over extended periods feasible. The results of our study illustrate the successful integration of the microfluidic platform and FD-FLIM for a single-spheroid oxygen consumption analysis, offering valuable insights into cellular physiology, especially in 3D in vitro models. The ability to perform single-spheroid oxygen consumption characterization using this developed approach provides a powerful tool for studying cellular physiology and metabolism under drug treatments, promising significant advancements in understanding cellular responses and drug effects.

## 2. Materials and Methods

### 2.1. Microfluidic Device Design and Fabrication

**Figure 1:**
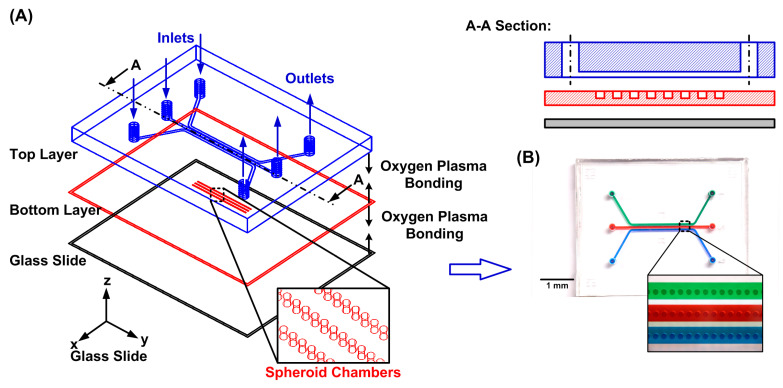
(**A**) Schematic of the microfluidic device for oxygen consumption characterization on single spheroids. (**B**) The experimental photos of the fabricated microfluidic device filled with food dyes.

To perform oxygen tension measurement in the surroundings of single spheroids, a microfluidic device is designed to aliquot groups of cells for spheroid formation, culture, and isolation in individual compartments in the experiments. The microfluidic device is made of an elastomeric material, polydimethylsiloxane (PDMS) (Sylgard 184, Dow Corning, Midland, MI, USA), due to its great optical transparency and manufacturability [18]. The device consists of two PDMS layers and a glass substrate as shown in Figure 1A. The top layer is a PDMS layer (c.a. 5 mm thick) with three microfluidic channels, and each channel is designed with an inlet and an outlet for the delivery of cells and reagents. Each channel can be used to test the different culture and treatment conditions on the formed spheroids on an identical chip for more consistent results and easier comparison. The channel is 600 and 300 μm in width and height, respectively, and the diameters of the inlet and outlet are 2 mm. In the bottom layer, a total of 150 circular compartments are designed with diameters and heights of 200 and 300 μm for spheroid formation and culture, respectively [19]. Each microfluidic channel on the top layer covers fifty compartments to aliquot groups of cells introduced into the channel. The bottom layer is designed with a thickness of 0.5 mm to minimize the effects of the oxygen absorption in PDMS on the measurements while keeping its great usability. In addition, a 0.33 mm thick glass slide is bonded underneath the bottom layer to minimize oxygen diffusion from the ambient.

The microfluidic device is fabricated using the well-developed soft lithography replica molding technique [20,21]. The master molds are prepared by patterning negative tone photoresists (SU-8 2100 and SU-8 2015, MicroChem Corp., Westborough, MA, USA) with the microfluidic channels or compartment geometries on silicon wafers using conventional photolithography. The molds are silanized using 1H,1H,2H,2H-perfluorooctyl trichlorosilane (91%) (L16606, Alfa Aesar, Ward Hill, MA, USA) in a desiccator at room temperature to prevent undesired bonding after PDMS curing. The PDMS prepolymer is prepared by mixing base and curing agent in a weight ratio of 10:1. The prepolymer is poured onto the fabricated mold and left inside an oven at 60 °C overnight to cure the top layer fabrication. After curing, the inlet and outlet of the top layer are punched using a biopsy punch. For the bottom layer, the PDMS precursor is spin-coated onto the mold at 100 rpm for 90 s resulting in a 0.5 mm thick bottom layer. Using an oxygen plasma surface treatment (90 W, 40 s), the thin bottom PDMS layer is first irreversibly bonded to the glass slide, and the assembled layer is irreversibly bonded to the thick top PDMS layer. The fabricated microfluidic device is kept inside the oven overnight at 60 °C to enhance the bonding strength between the PDMS layers and the thin glass slide. After fabricating the device, all the inlets and outlets are connected to cut 3 mL syringes through blunt needles as reservoirs for cell suspension and reagent delivery.

### 2.2. Cell Culture

To demonstrate the device’s capabilities for tumor spheroid formation and oxygen consumption analysis, human osteosarcoma cells (MG-63) obtained from Bioresource Collection and Research Center (BCRC, Hsinchu, Taiwan) are utilized for the cell experiments in this study. The stocks of MG-63 cells are cultured in growth medium composed of Minimum Essential Media (MEM) (Gibco 41090-036, Invitrogen Co., Carlsbad, CA, USA) with 10% v/v heat-inactivated fetal bovine serum (Gibco 10082, Invitrogen), 1% Antibiotic-Antimycotic (Gibco 15240, Invitrogen), 1% sodium pyruvate (Gibco 11360, Invitrogen), and 1% non-essential amino acids (Gibco 11140, Invitrogen). The cells are maintained under 5% CO_2_ in T25 cell culture flasks (Nunc 156367, Thermo Scientific Inc., Rochester, NY, USA), and passaged by dissociation with 0.25% trypsin–EDTA (Gibco 25200, Invitrogen) every three days. Cell suspensions for the experiments are made by centrifugation of the dissociated cells at 1000 rpm (209× *g*) for 5 min at room temperature. The culture medium is changed every other day during the experiments.

### 2.3. Tumor Spheroid Formation

**Figure 2:**
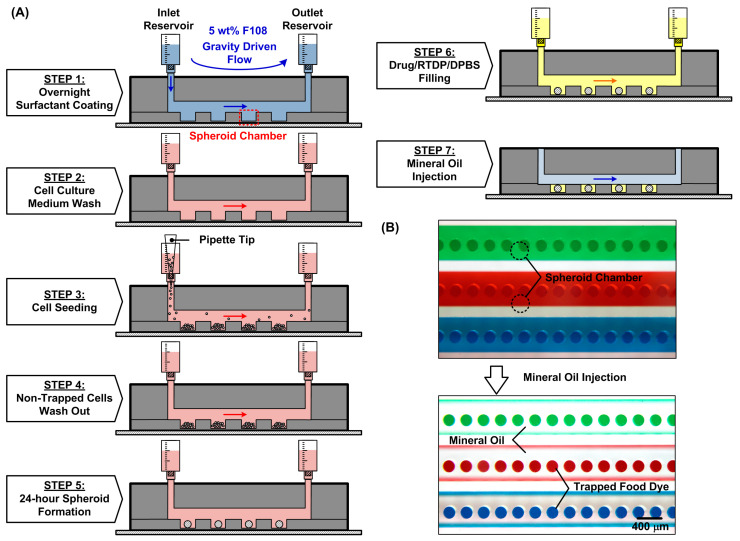
(**A**) Operation procedures of the microfluidic device to characterize oxygen consumption on the single spheroids. (**B**) Experimental photos of the device before and after the introduction of oil (STEP 7) to seal the spheroid chambers filled with food dyes.

In order to form the spheroids in the compartments and characterize their oxygen consumption, the entire experimental procedure is designed as shown in Figure 2. Before the experiments, the microfluidic channel walls are coated with 5 wt% surfactant (Synperonic F108, Sigma Aldrich 07579, Merck KGaA, Darmstadt, Germany) overnight to prevent cell attachment on the surfaces (STEP 1) [19]. The device is sterilized under UV light (center wavelength of 365 nm) for 2 h and left overnight with the surfactant; then, the surfactant is replaced by the cell culture medium (STEP 2) before the cell introduction. For spheroid formation, a 200 μL MG-63 cell suspension with a density of 1 × 10^6^ cells/mL is introduced into the devices which contain cell culture chambers 200 μm in diameter (STEP 3). In order to make the cells distribute uniformly across the entire microfluidic channel in the top layer, the cell suspension is introduced into the device with a flow rate greater than 100 μL/min [22]. After introducing the cells through the microfluidic channel, the seeded cells settle down in the culture chambers due to gravity and form 3D spheroids within 24 h in the absence of an adherent anchoring substrate. The entire cell trapping process lasts no longer than 20 min, including 10 min of channel filling with the cells followed by 5 min of static settlement and 5 min of washing away the non-trapped cells out of the device (STEP 4). After cell seeding, the device is kept in a humidified incubator with 5% CO_2_ at 37 °C. The medium in the device is exchanged every 12 h by adding 1 mL of fresh culture medium at the inlet and pipetting out the same amount of aged medium from the outlet as shown in Figure 2A. After 24 h, the spheroids are formed inside the microfluidic device (STEP 5). In order to observe the formed spheroids and characterize their sizes within devices, brightfield images of the spheroids are captured using an inverted microscope equipped with a CCD camera (ORCA-R2, Hamamatsu Photonics, Shizuoka, Japan).

### 2.4. Cell Viability

In order to confirm the cell compatibility of the device and cell viability under various culture conditions, fluorescence-imaging-based cell viability assays (LIVE/DEAD Viability/Cytotoxicity kit, L3224, Thermo Scientific Inc., Rochester, NY, USA) are performed in the experiments. In the assay, the live and dead cells are stained with calcein AM (2 μM) and ethidium homodimer-1 (EthD-1) (2 μM), respectively. The solution containing the live/dead staining dyes is introduced into the device and incubated in a cell incubator at 37 °C with 5% CO_2_ for 45 min to ensure the staining of the spheroids and the whole experimental procedure is protected from light. The staining solution is then replaced by the culture medium after staining to minimize the undesired fluorescence background during observation using an automated widefield inverted fluorescence microscope (DMi8, Leica Microsystems, Wetzlar, Germany). For quantification of the viability assay results, image analysis calculating cell viability by dividing the live cell number (the calcein-AM-stained cells) by the total cell number (stained cells) is performed on the captured fluorescence images. The analysis is performed using a computer code programmed on mathematics software (MATLAB 2017a, The MathWorks, Inc., Natick, MA, USA).

### 2.5. Oxygen Tension Measurement

For the characterization of oxygen tension in the surroundings of the spheroids in the compartments, the medium is replaced by Dulbecco’s phosphate-buffered saline, calcium, magnesium (DPBS) (Gibco 14040, Thermo Fisher Scientific Inc., Waltham, MA, USA) containing an oxygen-sensitive fluorescent dye, tris(2,2′-bipyridyl) ruthenium (II) chloride hexahydrate (RTDP) (50525-27-4, Sigma-Aldrich, St. Louis, MO, USA) (5 mg/mL) (STEP 6). It has been reported that the culture of cells in a medium containing low-concentration ruthenium-based oxygen-sensitive dye has minimum effects on cell viability [23]. Ruthenium-based oxygen-sensitive fluorescence dye has been broadly exploited for oxygen tension measurements due to its stability and desired relatively long fluorescence lifetime [16,24,25,26]. The ruthenium-based complex is a fascinating triplet emitter, and it relies on fluorescence quenching by oxygen and has been used in both intensity and lifetime-based measurements. Oxygen is known to be an effective collision quencher which reduces fluorophore yield resulting in a decrease in emitted fluorescence intensity as well as a reduction in fluorophore excited-state lifetime [27].

After the spheroid formation and oxygen-sensitive dye replacement, the syringe connecting to the channels is removed. The microwells are then isolated and sealed by injecting the mineral oil with low oxygen diffusivity and solubility (MitoXpress mineral oil MO-200L-1, Agilent Technologies, Inc., Santa Clara, CA, USA) into the channel from the inlet (STEP 7). The mineral oil acts as a barrier and prevents ambient oxygen diffusion into the microwells for accurate single-spheroid oxygen consumption (OC) measurement, as shown in Figure 2B. The oxygen tension can be quantitatively estimated by measuring the lifetime of the dye inside the chambers according to the Stern–Volmer equation. The quantitative oxygen tension can be calculated by

(1) $$\tau_{0}=\tau \cdot \left(1+K_{q}\left[O_{2}\right]\right)⟹\left[O_{2}\right]=\frac{1}{K_{q}}\left(\frac{\tau_{0}}{\tau }-1\right),$$

where $\tau_{0}$ and τ are fluorescence lifetimes without and with the presence of oxygen, respectively, and *K_q_* is a quenching constant.

To measure the fluorescence lifetime, a widefield frequency domain fluorescence imaging microscopy (FD-FLIM) setup based on a commercially available inverted fluorescence microscope (DMI 6000B, Leica Microsystems, Wetzlar, Germany) is used in the experiments. The detailed setup, characterization, and analysis process are described in previous literature [16,28]. Briefly, the microscope is equipped with a 470 nm LED (M470L3-C2, Thorlabs, Newton, NJ, USA) as a light source, and a high-sensitivity FLIM CMOS camera (PCO.FLIM, Excelitas Technologies Corp., Waltham, MA, USA) as a detector. The LED is powered by an LED driver (DC2200, Thorlabs) that is synchronized with the camera supporting a reference signal. The excitation and emission light signals are directed onto and collected from the samples through an objective (11506303, N Plan 5X/NA0.12 PH 0, Leica Microsystems) with a filter cube (11513808, I3 Filter Cube, Leica Microsystems). The camera recording parameters are set as 250 kHz, 500 ms, and 16 steps for the modulation frequency, exposure time, and phase steps, respectively, for the FLIM measurements. The setup provides the lifetime measurement with less than 5 ns deviation across the field of imaging. The FD-FLIM setup provides rapid and accurate oxygen tension measurement capability without tedious calibration processes. In the measurement, the quenching constant is first calibrated experimentally. Fluorescence images of the dye placed in atmospheric and nitrogen-flushed environments with oxygen tensions of approximately 20.9% and 0% are used as references with the fluorescence lifetime of 381 ns and 533 ns, respectively [16].

The fluorescence lifetime images of the dye filled in the microwells are captured for an hour-long period with an interval of 5 min. The quenching constant (*K_q_*) is then calculated based on the lifetime values measured using the FD-FLIM setup. The quenching constant can be calculated according to the equation derived from the Stern–Volmer equation as

(2) $$K_{q}=\frac{\tau_{N}-\tau_{A}}{\tau_{A}\cdot {\left[O_{2}\right]}_{A}}$$

The $\tau_{N}$ and $\tau_{A}$ are the fluorescence lifetime measured using the FD-FLIM setup when introducing pure nitrogen and the ambient air, respectively, and the ${\left[O_{2}\right]}_{A}$ is the oxygen tension measured on the dye placed in the atmospheric environment when introducing the air into the environmental chamber. The average oxygen tension value of the dye filled in the chamber when introducing air into the environmental chamber is then estimated based on the calculated quenching constant to check if the value is approximately 20.9% for validation of the quenching constant.

For the oxygen consumption measurement, the oxygen tension values within the spheroid chambers are monitored in a time-lapsed manner using the FD-FLIM setup. The fluorescence lifetime images of the dye filled in the chambers are captured for an hour-long period with an interval of 5 min right after the oil filling. In order to alleviate the inconsistency between the devices resulting from the optical setup variation, the spheroid chambers without spheroids in the same device at the same time point after the isolation process are exploited as references during the measurements. The average fluorescence lifetime value ($\tau_{E}$) measured on the dye filled in all the empty chambers is exploited as the lifetime value under the atmospheric oxygen condition (~20.9% O_2_) for the same device at the same time point. The oxygen tension values of the oxygen-sensitive dyes filled in the spheroid chambers with the spheroids can then be evaluated based on the aforementioned quenching constant (*K_q_*), the fluorescence lifetime within the spheroid chambers with (τ) and without the spheroids ($\tau_{E}$) according to the Stern–Volmer equation:

(3) $$\tau_{0}=\tau_{E}\cdot \left(1+K_{q}\cdot 20.9\%\right)⟹\left[O_{2}\right]=\frac{1}{K_{q}}\left(\frac{\tau_{0}}{\tau }-1\right).$$

### 2.6. Drug Treatment

In order to investigate the effects of drug treatment on the oxygen consumption of the MG63 spheroids, the spheroids are tested under two types of drugs: a chemotherapeutic drug, 5-fluorouracil (5-FU) (Sigma-Aldrich F6627, Merck KGaA, Darmstadt, Germany), and an antibiotic, oligomycin A (Sigma-Aldrich 75351, Merck). The two drugs have different modes of action that may affect the cellular metabolism. 5-FU, a commonly used anti-cancer drug, acts as a thymidylate synthase inhibitor which can block DNA replication and further affects cell division. In contrast, oligomycin is an ATP synthase inhibitor and can be used to prevent phosphorylating respiration of a cell.

In the drug treatment experiments, the chemotherapeutic drug 5-FU with a concentration of 250 µM in the growth medium is added to one of the channels with the spheroids cultured in the chambers, while the other channel with the untreated spheroids cultured in the chambers serves as the control. The spheroids are treated with 5-FU for 48 h, and the medium is replaced by Dulbecco’s phosphate-buffered saline, calcium, magnesium (DPBS) (Gibco 14040, Thermo Fisher Scientific Inc., Waltham, MA, USA) containing the oxygen-sensitive dye. The spheroids are then isolated by introducing mineral oil to cover the channel right before the oxygen tension measurement using the FD-FLIM setup. Similarly, after the formation of spheroids, the antibiotic oligomycin with a concentration of 5 µM in the growth medium is used to treat the MG63 spheroids and incubated for only 15 min. Following the 15 min incubation, the medium containing the drug is replaced by the aforementioned DPBS with the oxygen-sensitive dye, and the spheroids are then isolated using the mineral oil for the FD-FLIM-based oxygen tension measurement. Oxygen consumption of the single spheroids under both drug treatments is observed based on the measured lifetime values.

## 3. Results and Discussion

### 3.1. Formation and Culture of Single Spheroids

**Figure 3:**
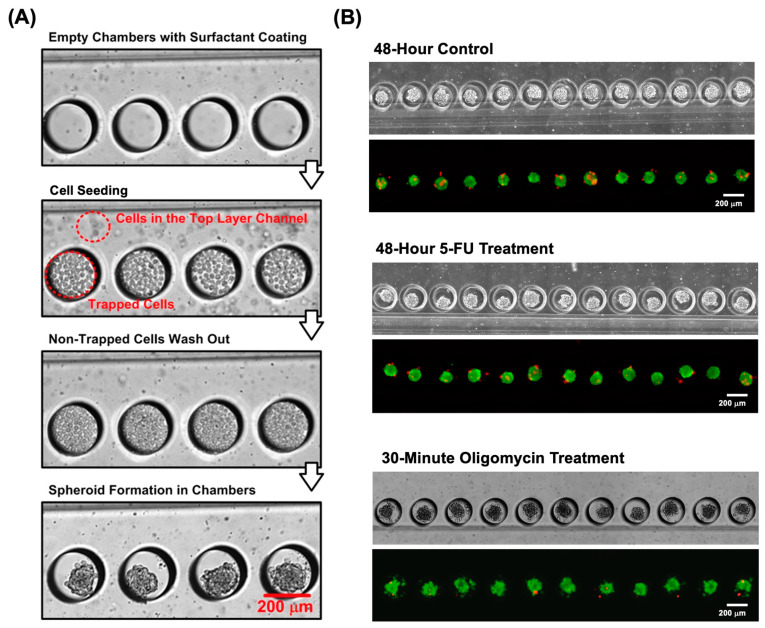
(**A**) Brightfield images of the devices during the cell seeding and spheroid formation processes. (**B**) Brightfield and fluorescent images of the spheroids cultured in the devices under control and drug treatment conditions. In the fluorescence images, the spheroids are fluorescently stained with calcein AM (green) and ethidium homodimer-1 (red) for live and dead cells, respectively.

In order to confirm the capability of the device for the formation and culture of the 3D single spheroids with controlled sizes, the MG63 cell suspensions with a cell density of 1 × 10^6^ cells/mL are tested in the experiments. The formation of the spheroids from the MG63 cells occurs spontaneously inside the microfluidic devices, as shown in Figure 3A. The images provide evidence of cellular aggregation and the subsequent formation of spheroids within a 24 h period. In order to investigate the spheroid size controllability using the devices, the spheroid size distributions in different microfluidic devices with the same cell seeding and culture conditions are analyzed. In the experiments, on the same device, the spheroids cultured in one of the side microfluidic channels are treated with a specific drug, and the spheroids cultured in another side channel remain untreated as a control group while the central channel is intentionally left empty to serve as an empty reference. Additionally, in order to confirm the consistent results obtained using the designed microfluidic devices, we compared the sizes of the spheroids formed in three separate devices in all the cell experiments.

### 3.2. Cell Viability and Spheroid Growth Analysis

To first confirm the compatibility of the device for spheroid culture, the fluorescence cell viability assay was performed in the experiments. Figure 3B displays the brightfield and fluorescent images of the MG63 spheroids fluorescently stained with the cell viability kit (calcein AM/ethidium homodimer-1) under different drug-treated and untreated conditions. It is noted that cell viability was performed on the cells treated with oligomycin for 30 min to ensure the live conditions of the cells during the experiments. The images show that the majority of the MG63 cells are live and show green fluorescence, which indicates that the cells can maintain great viability after 48 h of spheroid culture. In the analysis, the images were first subjected to contrast enhancement to optimize the visual clarity. Following this, segmentation techniques, notably thresholding, were applied to distinguish the spheroids from the background effectively. The resulting segmented images were processed to quantify the cell viability. Furthermore, the growth of the spheroids formed within the devices was also monitored by estimating the spheroid size change during the culture period under various conditions. The size of the spheroid was quantitatively estimated from the captured brightfield images, as shown in Figure 3B.

**Figure 4:**
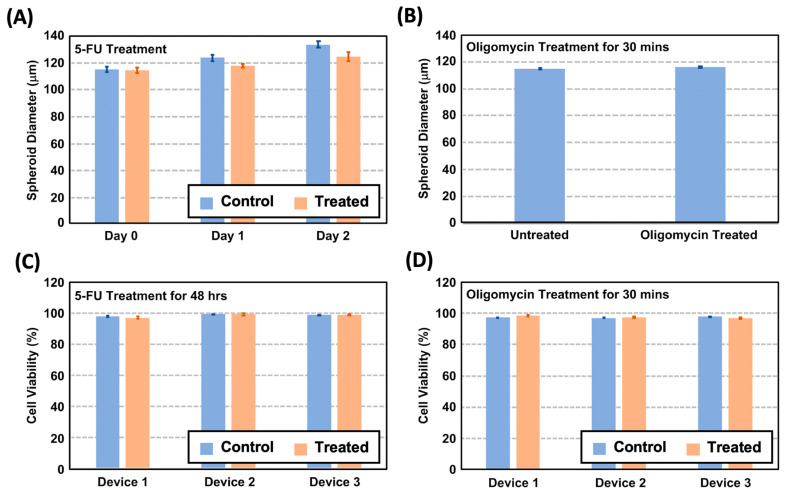
(**A**) The spheroid size variation in the untreated and 5-FU-treated spheroids from Day 0 to 2. (**B**) The spheroid size discrepancy of the untreated and treated spheroids after the oligomycin treatment. (**C**) Quantitative cell viability estimated from the fluorescent cell viability images on the spheroids cultured in different devices after the 48 h 5-FU treatment. (**D**) Quantitative cell viability estimated from the fluorescent cell viability images of the spheroids cultured in different devices after the 30 min oligomycin treatment.

In terms of the spheroid size, Figure 4A,B illustrate the average size distribution of the spheroids cultured in three different devices. The average diameters of the untreated spheroids formed and cultured within the 200 μm diameter spheroid chambers are approximately 114.9, 123.7, and 133.7 μm on Day 0, 1, and 2, respectively. In comparison, the spheroids treated with the chemotherapeutic drug 5-FU exhibit diameters of 114.3, 117.8, and 124.7 μm on the same respective days without statistical difference from the untreated ones. These results indicate that 5-FU, a chemotherapeutic agent, slightly slowed down the spheroid growth. The other drug, a metabolism inhibitor drug, oligomycin, was administered for a brief duration of only 15 min due to its rapid effectiveness as an ATP synthase inhibitor [29]. No significant difference in the spheroid size was observed due to the limited incubation time. The results show that our microfluidic device has the capability to form spheroids with precise size control. This enables the observation of the temporal growth of spheroids while maintaining their uniform sizes under consistent physiological conditions. The cultured samples hold promising potential for utilization in a variety of biological assays.

In terms of the cell viability, Figure 4C shows the cell viability analysis results of the 5-FU-treated and untreated MG63 spheroids cultured for 48 h in the devices. The results suggest that both the treated and untreated spheroids have viabilities greater than 98% after 48 h of treatment, indicating that the drug concentration used is capable of affecting the spheroid growth without greatly affecting the cell viability inside the microfluidic devices. Similarly, the cell viability analysis was performed for the oligomycin-treated spheroids, and the results are compared to the untreated spheroids. The result as shown in Figure 4D suggests that both the treated and untreated ones have viabilities greater than 97%, indicating that the spheroids can be grown well.

Since the oxygen consumption of the cells highly depends on their healthiness, the viability of the spheroids cultured in DPBS with the oxygen-sensitive dye is also evaluated based on the fluorescence staining. To confirm the cell compatibility of the MG63 cells with the oxygen-sensitive dye, we performed a cell cytotoxicity assay over 24 h using different concentrations of RTDP from 0.5 to 5 mg/mL on the MG-63 cells cultured in the conventional monolayer format based on our previously developed method. The result (Figure S1 in the Supplementary Material) shows that the relative cell viability is more than 85% without statistical difference from the control experiments, indicating the oxygen-sensitive dye is cell-compatible for all the tested concentrations. For the optimized measurement performance, the RTDP with a concentration of 5 mg/mL is used for the single-spheroid oxygen consumption measurement in the experiments.

### 3.3. Oxygen Tension Measurement in the Microfluidic Device

**Figure 5:**
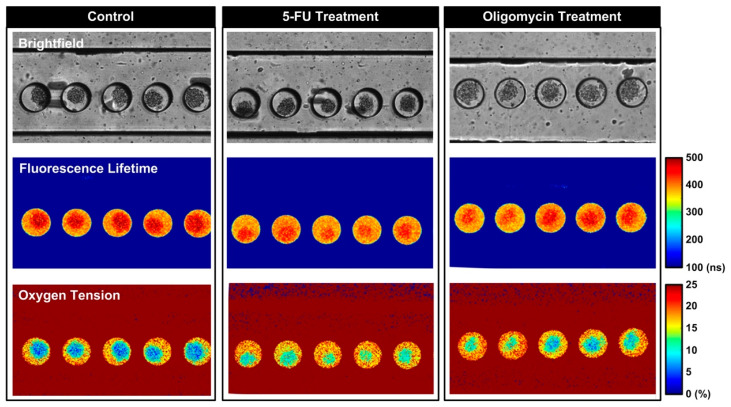
Brightfield, fluorescence lifetime, and the estimated oxygen tension images of the MG-63 spheroids cultured in the microfluidic devices under different conditions.

In order to estimate the oxygen consumption of the single spheroids, the FD-FLIM setup is employed to measure the fluorescence lifetimes of the oxygen-sensitive dye inside the chambers. Using an automated computer code, the lifetime values are calculated and converted to oxygen tension according to Equation (3). Figure 5 shows the brightfield, fluorescence lifetime, and calculated oxygen tension images of the spheroids formed and cultured within the microfluidic devices with different treatments at the end of the 60 min measurements. The images show that the fluorescence lifetime can be successfully measured using the FD-FLIM setup in the experiments, and the oxygen tensions within the chambers with single spheroids can be accurately estimated. In addition, the images show the oxygen gradients established within the spheroids can be observed using FD-FLIM, and the observation agrees with that reported in a previous study [30]. The results suggest that FD-FLIM can be exploited to investigate the spatial oxygen tension distribution within the three-dimensional spheroid culture in a non-invasive manner in the future.

**Figure 6:**
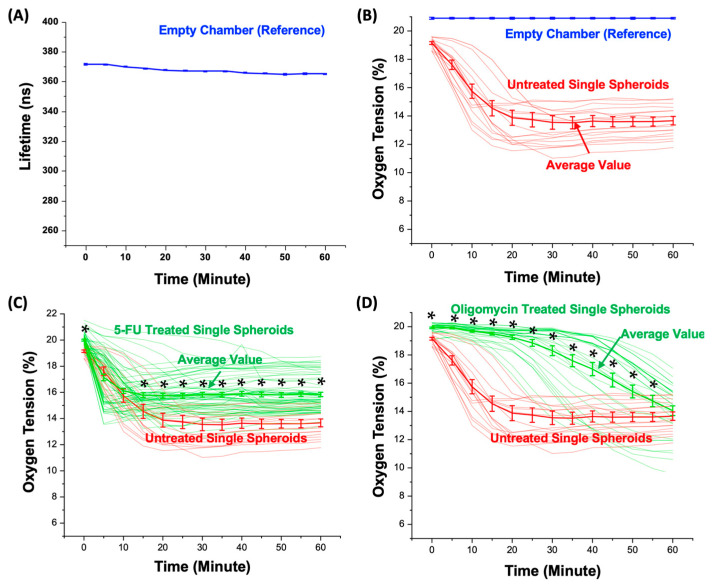
(**A**) Measured fluorescence lifetime (average value: 367.3 ns) within the spheroid chambers during the 60 min measurement. (**B**) The oxygen tension values estimated from the measured fluorescence lifetime within the spheroid chambers without and with untreated spheroids. The plots show all the oxygen tension variation curves measured from the single spheroids, and the average values presented as mean ± STD. (**C**) All the oxygen tension values estimated from the measured fluorescence lifetime within the spheroid chambers with the untreated and 5-FU-treated spheroids, and the average values (mean ± STD) during the measurement period. (**D**) All the oxygen tension values estimated over the measured fluorescence lifetime within the spheroid chambers with the untreated and oligomycin-treated spheroids, and the average values (mean ± STD) during the measurement period. (*: *p* < 0.05 for Student’s *t*-test).

Figure 6A shows the average calculated lifetime of the empty chambers without spheroids for an hour-long measurement period. The results indicate that the lifetime values of the empty chambers are between 370.0 and 373.1 ns with an average value of 371.3 ns at the beginning of the experiment, and slightly decrease to 363.6 to 366.5 ns with an average value of 364.9 ns after an hour-long time-lapsed measurement for every 5 min. The less than 1.7% lifetime variation suggests the consistency of the lifetime measurement. Without additional oxygen supply, the slight shortening of the fluorescence lifetime of the dye within the empty chambers may result from the quenching effect due to the intensive light exposure on the small amount of the dye (the volume is approximately 9.4 nL) filling the chambers during the one-hour measurement period. To compensate for this quenching effect, the average lifetime value measured on the empty microwells is exploited as the reference lifetime under the normoxia condition (20.9% O_2_).

### 3.4. Oxygen Consumption of the Single Spheroids

The average oxygen tension values are calculated across all the spheroid chambers to measure the oxygen consumption of the single spheroids cultured in the spheroid chambers. The oxygen tension variations and the average values of all the measured untreated spheroids during the 60 min measurement period are plotted in Figure 6B. The results show that the average oxygen within the microwells containing the untreated single spheroids decreases rapidly from 19.2% to 13.9% during the first 25 min of the measurement and maintains at a stable level between 13.7 and 13.8% during the last 30 min of the measurement. In addition, the measurement results show the heterogeneity in the oxygen consumption among the measured single spheroids, indicating the importance of the measurements at the single-spheroid level.

5-FU Treatment: Figure 6C shows the temporal average oxygen tension variations within each chamber with a single spheroid without or with exposure to the chemotherapeutic drug 5-fluorouracil (5-FU). As a chemotherapeutic agent, 5-FU is known to interfere with DNA and RNA synthesis, resulting in cell cycle arrest and the induction of apoptosis in rapidly dividing cancerous cells. The observations at various time points show remarkable alterations in the oxygen tension and provide valuable insights into the dynamic cellular responses to the drug. At the onset of the experiment (0 min), the chambers with the untreated spheroids exhibit oxygen tensions ranging from 18.5% to 19.7%, with an average value of 19.2%. The chambers with the 5-FU-treated spheroids display slightly a higher oxygen tension ranging from 18.8% to 21.5%, with an average value of 19.9%. During the first 10 min of the measurements, the oxygen tensions decrease in a similar manner for the chambers with both the untreated and 5-FU-treated spheroids without a statistical difference (*p* value greater than 0.05). However, after 15 min, the average oxygen tension within the chambers with the untreated spheroids settled on a lower oxygen tension level ranging from 14.7 to 13.8% (average: 13.9%) compared to those with the 5-FU-treated ones ranging from 15.7 to 15.8% (average: 15.8%). Subsequently, at the 60 min mark, the oxygen tension within each chamber with the untreated single spheroids ranges from 11.7% to 15.7%. In contrast, the oxygen tension within each chamber with the treated single spheroids displays a range between 13.3% and 18.7% with a statistically significant difference (*p* value lower than 0.05).

The results indicate that the chemotherapeutic drug can alter the oxygen consumption, leading to a discrepancy between the oxygen tension variation measured from the 5-FU-treated and untreated spheroids [4]. The initial oxygen tensions are similar right after the chambers are sealed using the mineral oil (0 min) for the spheroids. A pivotal shift in the oxygen tension dynamics became apparent as time progressed. At 15 min, the chambers with the untreated spheroids stabilized at a lower oxygen tension level (average 14.7%), while those with the 5-FU-treated spheroids maintained a higher tension level (average 15.7%). The results suggest that the spheroids responded to the chemotherapeutic drug 5-FU by altering their metabolism. In addition, there is a higher deviation in the measurement of the chambers with the 5-FU-treated spheroids compared to the untreated spheroids. The result suggests that the heterogeneity in the cellular response may be raised when the cancer cells face treatment stress, especially metabolism alteration in response to drug exposure. This variation emphasizes the importance of characterizing single spheroid responses to better understand the complexities of drug-induced effects.

The stable oxygen tension values can be observed in both the untreated and 5-FU-treated spheroids, maintaining a 1% difference from 15 to 60 min. The discrepancy is attributed to the limited oxygen and nutrient availability within the chambers. Furthermore, the reduction in the size of the 5-FU-treated spheroids is observed on Days 1 and 2, as depicted in Figure 4A, with a significant size difference of approximately 5.9 and 9.0 μm, respectively. The observation indicates the potent inhibitory effect of 5-FU on spheroid proliferation. This inhibitory impact is associated with the drug’s ability to block DNA replication and affect cell division. In addition, it has been reported that 5-FU disrupts the outer layer of the spheroids over time, while the core remains unaffected [31]. It is plausible that the 5-FU-treated spheroids exhibit altered metabolic demands, leading to a balance between oxygen consumption and supply. This finding may also indicate a potential shift towards anaerobic metabolism in response to drug-induced metabolic changes. [4] The dynamic nature of these changes suggests a complex interplay between the drug’s effects on the spheroid size and its influence on cellular metabolism. These findings contribute to a deeper understanding of the molecular and phenotypic changes induced by 5-FU in three-dimensional cell cultures, providing insights that may have implications for cancer therapeutics and drug development.

Oligomycin Treatment: Figure 6D shows the calculated oxygen tension within each chamber containing single spheroids, without or with exposure to an antibiotic, oligomycin A. As a metabolic inhibitor drug, oligomycin inhibits ATP synthase by blocking the proton channel, which is required for the oxidative phosphorylation of ADP to ATP (energy production), resulting in the prevention of cell respiration [32]. The observations at different time points reveal significant changes in the oxygen tension, offering valuable insights into the dynamic cellular responses triggered by the drug. At the onset of the experiment (0 min), the chambers with the untreated spheroids exhibit oxygen tensions ranging from 18.5% to 19.7%, with an average value of 19.2%. The chambers with the oligomycin-treated spheroids display a slightly higher oxygen tension ranging from 19.1% to 20.2%, with an average of 19.9%. During the first 20 min of the measurements, the oxygen tensions decrease rapidly from 19.1% to 13.8% for the chambers with the untreated spheroids. In contrast, the oligomycin treated-spheroids maintain a stable oxygen tension of 19.9% to 19.2% (average: 19.6%). After 20 min, the average oxygen tension within the chambers with the untreated spheroids settled on a lower oxygen tension level ranging from 11.5% to 19.2% (average: 13.9%). In comparison, after 20 min, the oligomycin-treated spheroids exhibited oxygen tension values within the range of 16.6% to 20.0% (average: 19.2%). Subsequently, at the 60 min mark, the oxygen tension within each chamber with the untreated spheroids ranged from 11.7% to 15.7% (average: 13.8%). In contrast, the oxygen tension within each chamber with the treated spheroids displayed a range between 9.5% and 17.0%, with an average of 14.0% without a statistical difference.

The results indicate that the metabolic inhibitor drug can result in the inhibition of oxygen consumption by cells, leading to a discrepancy between the oxygen tension variation measured from the untreated and oligomycin-treated spheroids [32]. The initial slow decrease in the oxygen tension within the oligomycin-treated spheroids right after the chambers are sealed using the mineral oil (0 min) indicates an immediate response to the drug, potentially in early cellular metabolic changes. A pivotal shift in oxygen tension dynamics became apparent as time progressed. From 0 to 20 min, the substantial difference in the oxygen tension observed between the untreated and treated spheroids emphasizes the rapid impact of oligomycin in inhibiting cellular respiration by blocking the proton channel, resulting in preventing oxygen consumption by the cells. At 20 min, the chambers with the untreated spheroids stabilized at a lower oxygen tension level (13.9%), while those with the oligomycin-treated spheroids maintained a higher tension level (19.2%). In addition, the oligomycin-treated spheroids show a large deviation after 20 min compared to the untreated spheroids and the deviation increases over time. At 60 min, the variation was around 9.5% to 17%. The higher deviation in the measurement of the chambers with the oligomycin-treated spheroids compared to the untreated spheroids may result from the cell heterogeneity and varied cell metabolism changes in response to the drug. The results suggest that the treated spheroids undergo metabolic changes in order to adapt to the microenvironment inside the chamber. The trend observed from 20 to 60 min shows that despite a decrease in the average oxygen tension, the oligomycin-treated spheroids maintain a more stable range of values, consistently maintaining a higher oxygen tension than the untreated spheroids showing delayed oxygen consumption effects, as reported by S. Russell et al. [4]. It is plausible that the spheroids initially adapt their metabolic processes or resort to alternative energy sources in response to the drug and adapt to the microenvironment with limited nutrients and oxygen supply, leading to the observed oxygen tension fluctuations.

The experimental results suggest that our developed approach, incorporating a microfluidic device and FD-FLIM setup for oxygen consumption characterization in single spheroids, demonstrates both precision and sensitivity in assessing the cellular metabolic dynamics of the spheroids. In three-dimensional cell spheroids, various gradients can be established, and the gradients can better represent the in vivo microenvironments within the tissues. The tested drugs may affect the single-cell metabolism as well as the spheroid structure and further alter the entire spheroid oxygen consumption estimating its metabolism. In order to better elucidate the mechanisms of the observed oxygen consumption change, more experiments and analysis is required in future research. Additionally, the development of an oxygen-sensitive dye with better stability and higher fluorescence/phosphorescence efficiency has the potential to enhance the capability of our approach to investigate the various transient metabolic activities of cells.

## 4. Conclusions

In this paper, an approach integrating a microfluidic device and FD-FLIM is developed to perform a single-spheroid oxygen consumption measurement with great throughput in a straightforward manner without tedious operation. The experimental results confirm the performance of the microfluidic device for highly efficient controlled spheroid culture and isolation in microwells. Furthermore, through using the FD-FLIM with an oxygen-sensitive phosphorescent dye, the oxygen tension variation within the microwells can be reliably, quantitatively, characterized in a time-lapsed and high-throughput manner for the oxygen consumption estimation. The experimental results show great sensitivity, and the measured average oxygen consumption value of a spheroid is similar to those obtained from the literature based on measurements using a commercially available instrument [4]. The ability of the developed approach to estimate the oxygen consumption of spheroids will help us to understand spheroid metabolism and its response to various drugs. Moreover, the approach can be further applied to study the transient oxygen consumption responses of spheroids in well-controlled microenvironments established inside microfluidic devices mimicking various in vivo conditions. This also has significant implications for personalized medicine and the reduction in animal testing reliance in biomedical research, ultimately leading to improved patient outcomes and scientific advancements.

## Data Availability

The data that support the findings of this study are available from the corresponding author upon reasonable request.

## Supplementary Materials

The following supporting information can be downloaded at https://www.mdpi.com/article/10.3390/bios14020096/s1, Figure S1: Cytotoxicity of the RTDP in DPBS for the MG-63 cells cultured as monolayers.
