# Supplementary material for: Characterization of Single-Spheroid Oxygen Consumption Using a Microfluidic Platform and Fluorescence Lifetime Imaging Microscopy
Source: Biosensors (Basel). 2024 Feb 11;14(2):96. doi: 10.3390/bios14020096 (PMC10887112; doi:10.3390/bios14020096)
Supplement: Supplementary file 1 [file biosensors-14-00096-s001.zip › biosensors-2845817-supplementary.pdf]

biosensors

Supplementary Materials

Characterization of Single-Spheroid Oxygen Consumption Using a Microfluidic Platform and Fluorescence Lifetime Imaging Microscopy

Santhosh Kannan [1,2,3], Chien-Chung Peng [1], Hsiao-Mei Wu [4] and Yi-Chung Tung [1,5,*]

1 Research Center for Applied Sciences, Academia Sinica, Taipei 115201, Taiwan
2 Department of Engineering and System Science, National Tsing Hua University, Hsinchu 300044, Taiwan
3 Nano Science and Technology Program, Taiwan International Graduate Program (TIGP), Academia Sinica, Taipei 115201, Taiwan
4 Department of Biomechatronics Engineering, National Taiwan University, Taipei 106319, Taiwan
5 College of Engineering, Chang Gung University, Taoyuan 33302, Taiwan
* Correspondence: tungy@gate.sinica.edu.tw; Tel.: +886-2-2787-3138

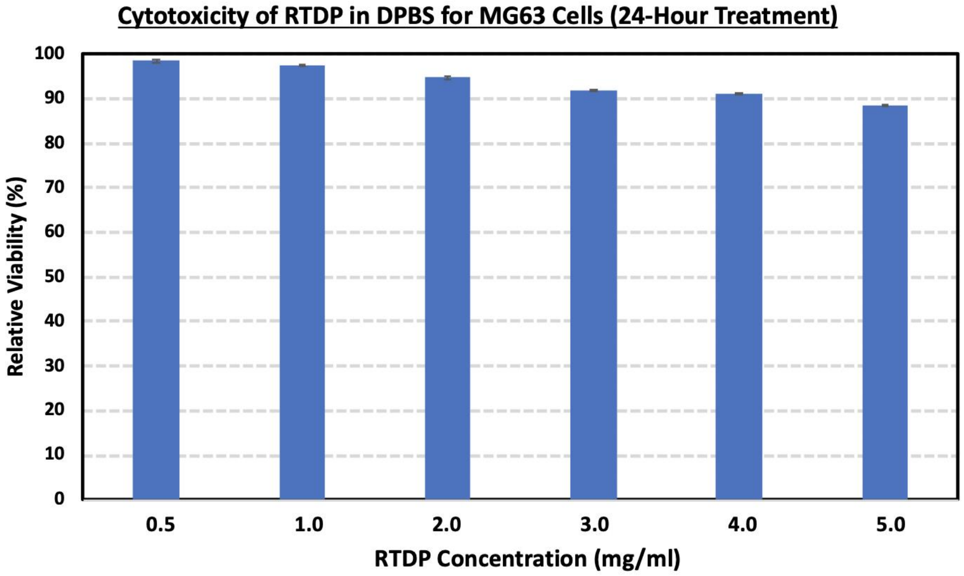

Figure S1. Cytotoxicity of the RTDP in DPBS for the MG-63 cells cultured as monolayers.
